# Comment on De Rosa et al. Hearing Loss: Genetic Testing, Current Advances and the Situation in Latin America. *Genes* 2024, *15*, 178

**DOI:** 10.3390/genes15111401

**Published:** 2024-10-30

**Authors:** Ana Belén Elgoyhen, Paula Inés Buonfiglio, Viviana Dalamón

**Affiliations:** 1 Laboratory of Physiology and Genetics of Hearing, Instituto de Investigaciones en Ingeniería Genética y Biología Molecular “Dr. Héctor N. Torres”, Consejo Nacional de Investigaciones Científicas y Técnicas, INGEBI/CONICET, Vuelta de Obligado 2490, Ciudad Autónoma de Buenos Aires C1428ADN, Argentina; abelgoyhen@gmail.com (A.B.E.); paulabuonfiglio@gmail.com (P.I.B.); 2 Instituto de Farmacología, Facultad de Medicina, Universidad de Buenos Aires, Ciudad Autónoma de Buenos Aires C1121ABG, Argentina

The manuscript “Hearing Loss: Genetic Testing, Current Advances and the Situation in Latin America” by De Rosa et al. [1] includes a comprehensive wealth of data that describe the state of the field in the Latin American region. A detailed demographic and economic landscape is presented, and provides insightful historical perspectives on key genetic advancements in hearing loss (HL) diagnosis. Nevertheless, the paper lacks important additional information concerning the current situation of genetic and genomic medicine in Latin American countries. As presented in the manuscript, Latin America has limitations regarding molecular genetic diagnosis, mainly due to a lack of resources. However, the omission by De Rosa et al. of certain published papers gives rise to a prevailing sense of complete scarcity of professionals and specialized centers dedicated to the genetic diagnosis of hearing loss within the region. The review states that massive sequencing technologies are either not available or very limited in certain countries and that there is no access to trained professionals or specialized centers for these tools. In addition, De Rosa et al. indicate that access to genetic studies for patients, when performed, focuses on the two frequently altered genes *GJB*2 and *GJB*6, mainly due to the lack of access to other options in each country. Although this is true in certain areas of Latin America, in recent years, genetic screening has expanded to additional genes following international guidelines. In this comment, we aim to supplement the existing information by introducing pertinent data from public resources, thereby offering the reader a more comprehensive understanding of the situation in the region.

De Rosa et al. effectively documented that genetic studies in Latin America have confirmed that alterations in *GJB*2 are a frequent cause of non-syndromic HL. However, they also state that “In most regions, even basic screening like GJB2/GJB6 are not available…”. This assertion inaccurately diminishes the research conducted in the Latin American region, as De Rosa et al. highlight a variety of studies on *GJB*2 and *GJB*6. Furthermore, a previous review of Lezirovitz et al. about the genetic etiology of HL in Latin America [2] cited by the De Rosa et al. included a total of 88 publications, approximately half of which focused on *GJB*2-*GJB*6 analysis, demonstrating that it is a study widely performed in the region.

The authors did not reference recent regional studies on the analysis of alterations in the stereocilin (*STRC*) gene. Current data suggest that these alterations are the next most common genetic cause of hearing loss worldwide, accounting for approximately 30% of patients with mild to moderate hearing loss and 16% among all groups [3]. Most *STRC* variants are large copy number variants (CNVs) and are less studied due to the technical challenges of analyzing the genomic locus, which includes a segmental duplication with a nearly identical pseudogene [4]. This complexity requires complementary testing strategies and has led to specific testing protocols [5,6,7,8,9], which have been introduced in certain Latin American countries. Thus, a study from Brazil tested 100 patients for *STRC* CNVs, yielding negative results [10]. Nevertheless, an additional report on an unaffected cohort of 2097 individuals from Brazil revealed that heterozygous copy number variants in the *STRC* gene were among the top five most frequent alterations detected [11]. In Argentina, a diagnosis rate of 5.7% was reported upon analyzing *STRC* CNVs in a cohort of patients with moderate HL [12].

De Rosa et al. state that “While Next-Generation Sequencing (NGS) is widely utilized in developed nations for the molecular diagnosis of HL, its adoption in Latin America remains far from optimal…”. Although the use of NGS in Latin American countries is far behind that of developed countries, it is worth mentioning that the region is going in that direction, and whole-exome sequencing has been used to diagnose the genetic causes of hearing loss in patients. Table 1 includes reports of NGS in Latin American cohorts with HL not referenced by De Rosa et al.

We agree with De Rosa et al. that Latin America needs more resources and an increase in access to medical healthcare and personalized medicine. However, it is imperative to underscore the endeavors within the region aimed at enhancing genetic diagnosis, broadening the scope of tested genes, and acknowledging the proficiency and contributions of experts dedicated to ensuring that state-of-the-art technology is accessible to the population. Although there is still a long road ahead in order to bring genetic counseling and genetic/genomic studies to Latin Americans, some public entities are making progress in this area (Table 2).

## Figures and Tables

**Table 1:** NGS analysis performed in Latin American countries.

Country	Study	Number of Tested Patients	Diagnosis Rate (%)	Reference
Argentina	WES	32	62.5	[13]
Brasil	NGS	81	24–58	[14,15,16,17]
México	WES	72	25–74	[18,19]
Cuba	NGS	11	100	[20]

**Table 2:** Centers dedicated to the study of the genetics of hearing loss in Latin America.

Country	Institute	Website
Argentina	National Center of Medical Genetics—ANLIS MALBRÁN	http://www.anlis.gov.ar/cenagem/?page_id=390 (accessed on 12 July 2024)
Argentina	Laboratory of Physiology and Genetics of Hearing, INGEBI-CONICET	https://ingebi-conicet.gov.ar/es_fisiologia-y-genetica-de-la-audicion/ (accesed on 12 July 2024)
Argentina	S.A.M.I.C Pediatric Hospital “Prof. Dr. Juan P. Garrahan”	https://www.garrahan.gov.ar/equipos-y-especialidades/servicios (accesed on 12 July 2024)
Argentina	CEPIDEM—University of Medicine	https://cepidem.sitios.fcm.unc.edu.ar/investigacion/ (accesed on 12 July 2024)
Brasil	Ministry of Health, Unified Health system	(https://www.gov.br/saude/pt-br/assuntos/noticias/2022/novembro/diagnostico-precoce-e-acompanhamento-reduzem-casos-de-surdez (accesed on 12 July 2024)
Brasil	Institute of Biosciences, Department of Genetics and Evolutionary Biology, University of Sao Paulo.	https://www.ib.usp.br/genetica-e-biologia-evolutiva# (accesed on 12 July 2024)
Brasil	Center for Molecular Biology and Genetic Engineering, Public University in Campinas.	https://www.cbmeg.unicamp.br/laboratorios/laboratorio-de-genetica-molecular-humana/ (accesed on 12 July 2024)
Chile	University of Valparaíso	https://cinv.uv.cl/ (accesed on 12 July 2024)
Chile	Center of Cellular and Integrative Physiology, Faculty of Medicine, Clínica Alemana University of Development.	https://www.cbmeg.unicamp.br/laboratorios/laboratorio-de-genetica-molecular-humana/ (accesed on 12 July 2024)
